# Involvement of H2A variants in DNA damage response of zygotes

**DOI:** 10.1038/s41420-024-01999-0

**Published:** 2024-05-14

**Authors:** Yuan Wang, Dai Tsukioka, Shoji Oda, Masataka G. Suzuki, Yutaka Suzuki, Hiroshi Mitani, Fugaku Aoki

**Affiliations:** 1 Department of Computational Biology and Medical Sciences, The University of Tokyo, Kashiwa, Chiba 277-8562 Japan; 2 Department of Integrated Biosciences, Graduate School of Frontier Sciences, The University of Tokyo, Kashiwa, Chiba 277-8562 Japan

**Keywords:** Embryology, Mitosis

## Abstract

Phosphorylated H2AX, known as γH2AX, forms in response to genotoxic insults in somatic cells. Despite the high abundance of H2AX in zygotes, the level of irradiation-induced γH2AX is low at this stage. Another H2A variant, TH2A, is present at a high level in zygotes and can also be phosphorylated at its carboxyl end. We constructed H2AX- or TH2A-deleted mice using CRISPR Cas9 and investigated the role of these H2A variants in the DNA damage response (DDR) of zygotes exposed to γ-ray irradiation at the G2 phase. Our results showed that compared to irradiated wild-type zygotes, irradiation significantly reduced the developmental rates to the blastocyst stage in H2AX-deleted zygotes but not in TH2A-deleted ones. Furthermore, live cell imaging revealed that the G2 checkpoint was activated in H2AX-deleted zygotes, but the duration of arrest was significantly shorter than in wild-type and TH2A-deleted zygotes. The number of micronuclei was significantly higher in H2AX-deleted embryos after the first cleavage, possibly due to the shortened cell cycle arrest of damaged embryos and, consequently, the insufficient time for DNA repair. Notably, FRAP analysis suggested the involvement of H2AX in chromatin relaxation. Moreover, phosphorylated CHK2 foci were found in irradiated wild-type zygotes but not in H2AX-deleted ones, suggesting a critical role of these foci in maintaining cell cycle arrest for DNA repair. In conclusion, H2AX, but not TH2A, is involved in the DDR of zygotes, likely by creating a relaxed chromatin structure with enhanced accessibility for DNA repair proteins and by facilitating the formation of pCHK2 foci to prevent premature cleavage.

## Introduction

Maintaining genetic integrity is crucial during early embryo development, as DNA damage at this stage has far-reaching consequences, even affecting future generations. This is particularly true for 1-cell stage embryos, also referred to as zygotes, which are highly likely to transmit DNA damage to all of their descendant cells. Nevertheless, the mechanisms through which zygotes respond to DNA damage to safeguard genetic integrity remain a largely unexplored area of research.

Most studies on DNA damage response (DDR) have been focused on somatic cells. Among the various forms of DNA damage, DNA double-strand breaks (DSBs) pose severe threats to genomic stability when they remain unrepaired. In somatic cells, the MRE11-RAD50-NBS1 (MRN) complex binds to DSBs and recruits ataxia telangiectasia mutated (ATM), a critical kinase involved in response to DSBs, leading to the phosphorylation of many DDR-related proteins, including Η2ΑΧ, one of the earliest substrates targeted by ATM [1, 2]. H2AX contains an SQ motif at its carboxyl end, in which the serine residue undergoes phosphorylation [3]. The resulting phosphorylated H2AX, known as γH2AX, acts as a loading platform for additional ATM molecules to bind to the chromatin flanking the damaged site, thus creating a positive feedback loop that amplifies the DDR signals [4]. ATM also phosphorylates CHK2 in response to DSBs, leading to G2 arrest and providing time for DNA repair to occur [5]. H2AX deficiency impairs G2 arrest in response to low doses of radiation in somatic cells, likely because of its role in amplifying DNA damage signals [6]. However, when exposed to doses higher than 5 Gy, no difference in G2 arrest can be observed between wild-type and H2AX-deleted cells [6]. While the impact of H2AX deficiency on DDR and cell sensitivity has been extensively researched in somatic cells [6–8], it remains to be well elucidated in zygotes.

In a previous study, we systematically investigated the cell cycle checkpoints in mouse zygotes in response to 10 Gy γ-ray, which mainly induces DSBs. It was found that irradiated zygotes were arrested only in the G2 phase even when they were exposed to γ-ray in the G1 or S phase [9]. The lack of G1 and S checkpoints likely contributes to the maintenance of genetic integrity by eliminating embryos with potential genetic alterations and preventing the transmission of mutations to subsequent generations [10].

Histone variants undergo global reprogramming after fertilization, creating a unique chromatin context during the first cell cycle that potentially leads to distinctive DNA damage response [2]. The oocyte-specific H1foo is abundant in the chromatin of zygotes, whereas somatic-type linker histones, with the exception of H1A, are absent [11]. The H3 variants, H3.1 and 3.2, are scarcely deposited in the chromatin of zygotes, while H3.3 is prevalent [12]. H2A exhibits the highest diversity in variants among the four core histones [13]. Detailed RNA sequencing analysis reveals that TH2A is the most highly expressed H2A variant in GV oocytes and zygotes, with its RNA level four times higher than H2AX, the second highest variant, while macroH2A and H2AZ are present in low amounts at the RNA level (Fig. S1) [13, 14]. At the protein level, H2AX and TH2A are highly deposited in the nucleosomes of zygotes, while macroH2A and H2AZ are rarely detected [13, 15]. Despite the high abundance of H2AX in zygotes, γH2AX level after irradiation exposure is relatively low compared to germinal vesicle (GV) stage oocytes or later-stage embryos [16, 17]. Therefore, the requirement of H2AX for DDR in the first cell cycle remains unclear. TH2A is specifically expressed in germ cells and early-stage embryos [15, 18]. TH2A also has an SQ motif at its carboxyl end, but it is unclear whether this SQ motif is involved in DDR. Although it has been previously reported that TH2A is phosphorylated on the threonine residue following its SQ motif as zygotes enter the M phase in the first cell cycle [19], the involvement of TH2A phosphorylation in DDR has not been investigated.

In the present study, we generated mice lacking either H2AX or TH2A using CRISPR-Cas9 and investigated the role of these two H2A variants in the DDR of zygotes exposed to both low and high doses of irradiation during the G2 phase of the first cell cycle.

## Results

### DNA damage response is effective during the G2 phase of the first cell cycle

**Fig. 1:**
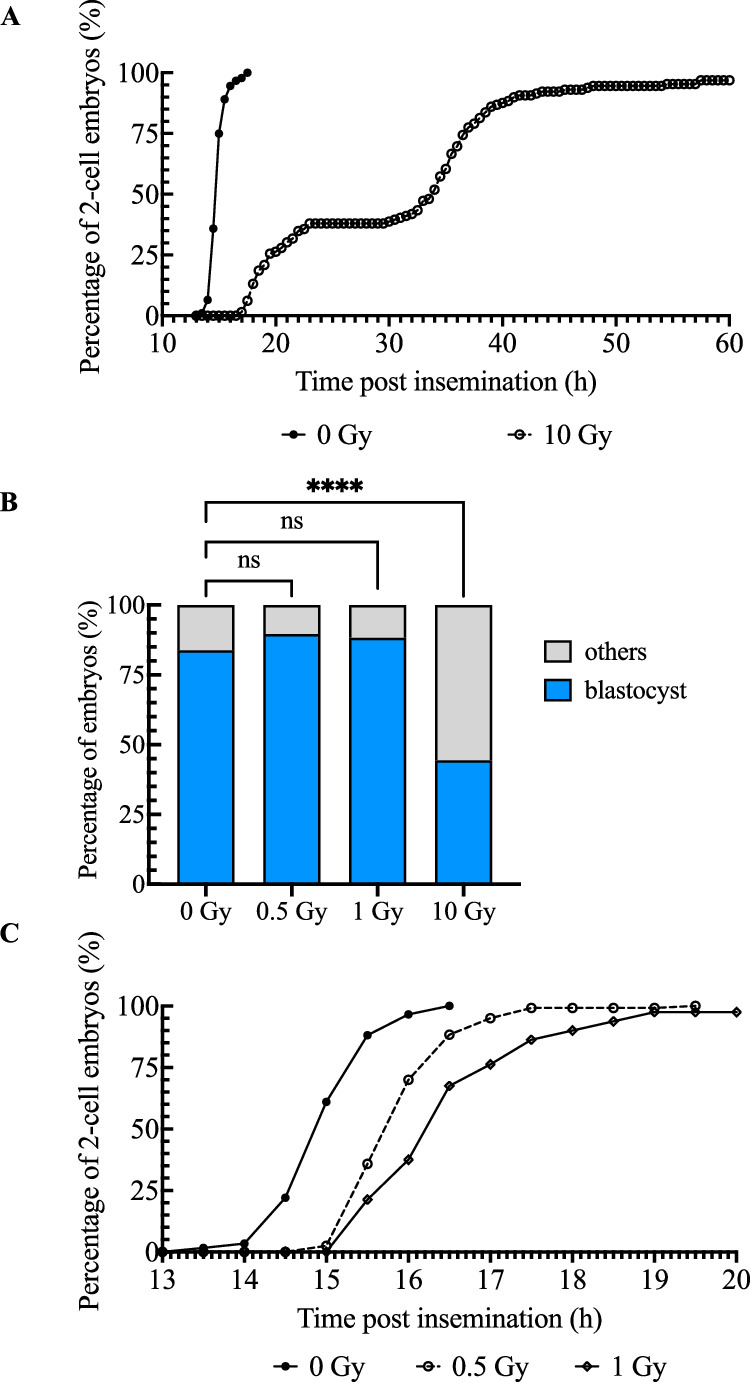
Effect of irradiation on G2 phase zygotes. **A** Cleavage curves of non-irradiated zygotes and zygotes exposed to 10 Gy irradiation at the G2 phase of the first cell cycle. The data represent the cumulative results of five independent experiments, examining a total of 92 and 129 embryos in the groups irradiated with 0 and 10 Gy, respectively. **B** Blastocyst formation rates of zygotes exposed to 0, 0.5, 1, or 10 Gy of irradiation at the G2 phase of the first cell cycle. The data represent the cumulative results of three independent experiments, examining a total of 74, 58, 60, and 54 embryos in the groups irradiated with 0, 0.5, 1, and 10 Gy, respectively. Fisher’s exact test was used for statistical analysis (****P < 0.0001; ns, not significant). **C** Cleavage curves of non-irradiated zygotes and zygotes exposed to 0, 0.5, or 1 Gy irradiation at the G2 phase of the first cell cycle. The data represent the cumulative results of five independent experiments, with a total of 118, 120, and 80 embryos examined for the groups irradiated with 0, 0.5, and 1 Gy, respectively.

Our previous research suggested that timely arrest of cell cycle progression following 10 Gy irradiation during the G2 phase of the first cell cycle was essential for embryos to repair DNA damage [9]. While utilizing 10 Gy irradiation offers the advantage of fully activating DDR and associated proteins and facilitating their detection, it is important to note that the extremely high level of DNA damage caused by such a high dose can exceed the repair capacity of zygotes and pose challenges in assessing DDR. Therefore, we also investigated the impact of lower doses, including 0.5 and 1 Gy irradiation, on preimplantation development. A live imaging device was used to record cell divisions, thereby avoiding interference with embryo culture and enabling the detection of transient cell cycle arrest. Consistent with our previous result, 10 Gy irradiation at the G2 phase severely delayed the first cleavage, which allowed > 40% of embryos to reach the blastocyst stage (Fig. 1A, B, Supplementary Movie 1). Zygotes irradiated with 0.5 Gy showed an approximately one-hour delay in cleaving into the 2-cell stage, and the cell cycle arrest in 1 Gy-irradiated zygotes was slightly longer (Fig. 1C, Supplementary Movie 2). As expected, the arresting times were proportional to the irradiation doses, given that higher doses cause more DNA damage, which takes a longer time to repair (Fig. 1A, C). Zygotes exposed to both lower doses subsequently developed to the blastocyst stage at a comparable rate to control embryos (Fig. 1B), suggesting that DDR in zygotes is functional and effective. Since the results of 0.5 and 1 Gy irradiation were similar and 0.5 Gy appeared sufficient to activate an observable cell cycle arrest, we chose to use 0.5 and 10 Gy in subsequent experiments.

### The absence of H2AX but not TH2A impairs the preimplantation development of irradiated zygotes

To investigate the role of H2AX and TH2A in the DNA damage response (DDR) of zygotes, we generated H2AX and TH2A knockout mice using CRISPR-Cas9 (Fig. S2A, B). Consistent with previous studies [8, 20], our H2AX-KO male mice were also infertile. As our RNA sequencing data have shown that both H2AX and TH2A in zygotes are maternally derived [14], we obtained zygotes deficient in H2AX or TH2A by crossing H2AX- or TH2A-KO female mice with wild-type male mice (Fig. S1C). Immunostaining confirmed the complete absence of H2AX or TH2A proteins in the resulting zygotes (Fig. S2D, E). Furthermore, TH2A was absent in embryos derived from TH2A-KO oocytes and wild-type sperm throughout the entire preimplantation period (Fig. S3A), indicating the full maternal origin of TH2A in zygotes. However, H2AX was found to be expressed at the 2-cell stage in embryos generated by H2A-KO oocytes and wild-type sperm (Fig. S3B). To further confirm the absence of H2AX at the entire 1-cell stage, the deposition of H2AX in M-phase zygotes was examined. Wild-type zygotes showed robust H2AX staining due to highly condensed chromosomes at the M phase, whereas H2AX was still not detected in the zygotes generated using H2AX-KO oocytes (Fig. S3C). Additionally, the increase in γH2AX was only observed in wild-type zygotes after 10 Gy irradiation (Fig. S3D). Therefore, the zygotes obtained, as illustrated in Fig. S2C, could be considered H2AX- or TH2A-deleted zygotes and used in subsequent experiments to investigate the involvement of H2AX or TH2A in the DDR of zygotes.

**Fig. 2:**
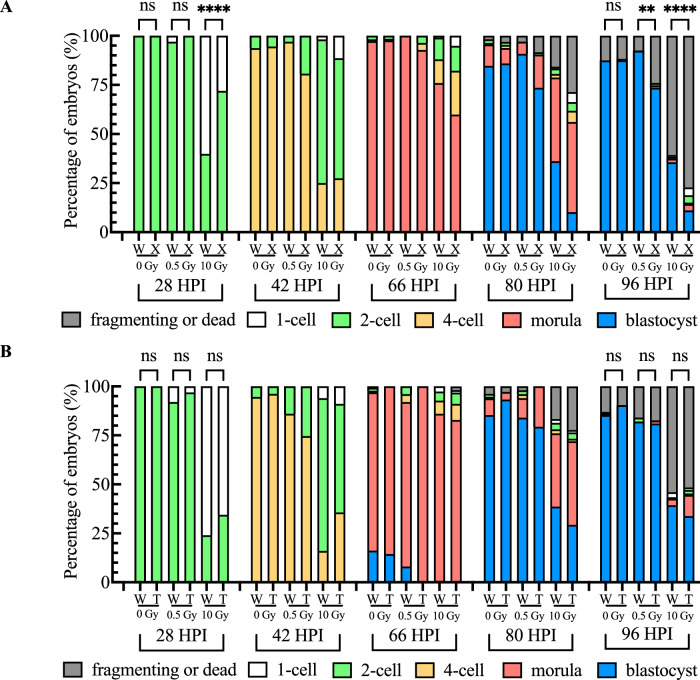
Preimplantation development of H2AX- or TH2A-deleted zygotes following irradiation exposure. **A** Preimplantation development of wild-type (W) and H2AX-deleted (X) zygotes irradiated with 0, 0.5, or 10 Gy at the G2 phase of the first cell cycle. More than four independent experiments were conducted for each experimental condition and the data represent the cumulative results. A total of 111, 65, and 108 embryos were examined for the wild-type groups irradiated with 0, 0.5, and 10 Gy, respectively. A total of 128, 83, and 157 embryos were examined for the H2AX-deleted groups irradiated with 0, 0.5, and 10 Gy, respectively. **B** Preimplantation development of wild-type (W) and TH2A-deleted (T) zygotes irradiated with 0.5 or 10 Gy at the G2 phase of the first cell cycle. More than four independent experiments were conducted for each experimental condition and the data represent the cumulative results. A total of 130, 50, and 150 embryos were examined for the wild-type groups irradiated with 0, 0.5, and 10 Gy, respectively. A total of 104, 63, and 157 embryos were examined for the TH2A-deleted groups irradiated with 0, 0.5, and 10 Gy, respectively. In both **A** and **B**, Fisher’s exact test was used for statistical analysis (**P < 0.01; ****P < 0.0001; ns, not significant).

We first compared the preimplantation development of wild-type and mutant embryos. Despite the abundant deposition of both H2AX and TH2A into the pronuclei of zygotes, the absence of H2AX or TH2A during the first cell cycle after fertilization surprisingly had no effect on preimplantation development of non-irradiated embryos (Fig. 2A, B). We then investigated the effect of H2AX or TH2A deletion on embryos under genotoxic stress induced by ionizing radiation. When zygotes were exposed to 10 Gy of irradiation, more than half of wild-type zygotes had not cleaved to the 2-cell stage by 28 HPI. Although TH2A deletion did not result in a significant change in the development to the 2-cell stage at 28 HPI, > 70% of H2AX-deleted zygotes progressed to the 2-cell stage (Fig. 2A, B). Furthermore, there was no significant difference in the blastocyst formation rate between wild-type and TH2A-deleted zygotes following irradiation (Fig. 2B). In contrast, H2AX-deleted zygotes showed a compromised ability to recover from irradiation-induced damage and had a much lower blastocyst formation rate than wild-type embryos exposed to both 0.5 and 10 Gy irradiation (Fig. 2A). These results indicate that H2AX, rather than TH2A, is involved in the DDR of zygotes.

### DNA damage-induced cell cycle arrest was shortened in H2AX-deleted zygotes exposed to both low and high-dose irradiation

**Fig. 3:**
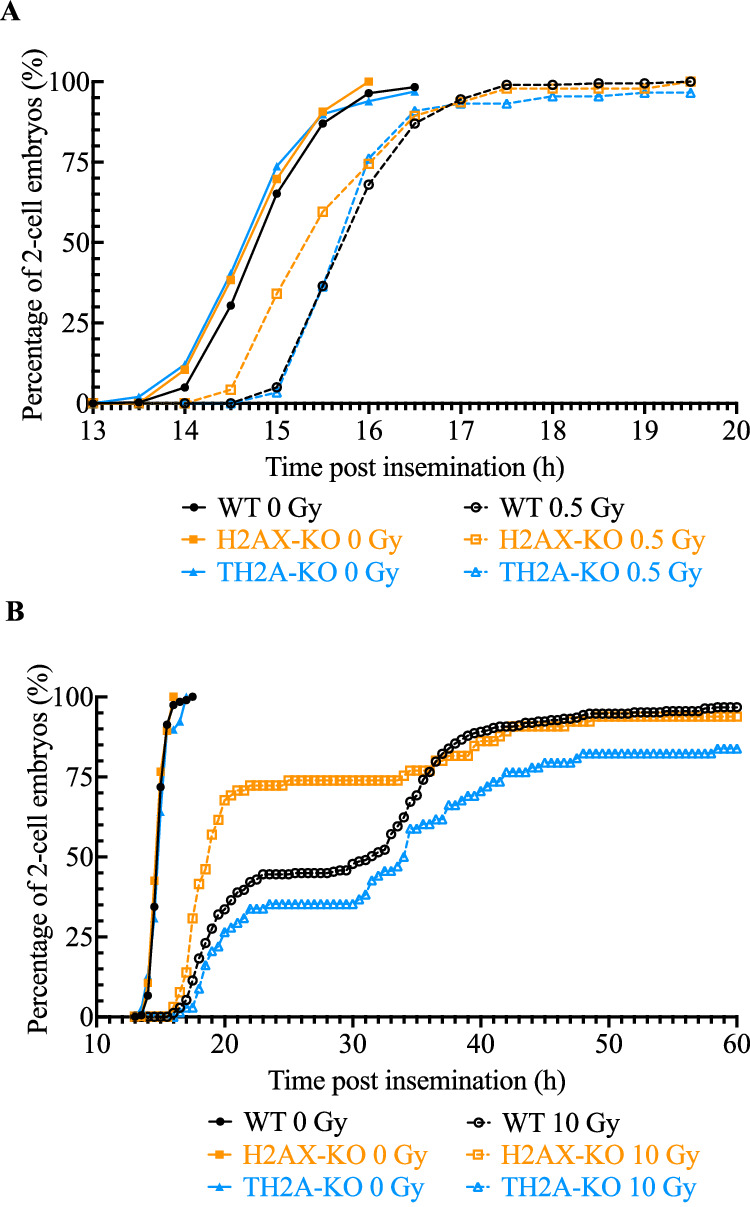
The first cleavage of wild-type (WT) and H2AX- or TH2A-deleted (XKO or TKO) zygotes following irradiation exposure. **A** Cleavage curve of WT, XKO, and TKO zygotes exposed to 0 or 0.5 Gy irradiation at the G2 phase of the first cell cycle. More than four independent experiments were conducted for each experimental condition and the data represent the cumulative results. A total of 197, 86, and 99 embryos were examined for WT, XKO, and TKO irradiated with 0 Gy, respectively. A total of 100, 94, and 88 embryos were examined for WT, XKO, and TKO irradiated with 0.5 Gy, respectively. **B** Cleavage curves of WT and H2AX- or TH2A-deleted zygotes exposed to 0 or 10 Gy irradiation at the G2 phase of the first cell cycle. More than four independent experiments were performed for each experimental condition and the data represent the cumulative results. A total of 195, 141, and 117 embryos were examined for WT, XKO, and TKO irradiated with 0 Gy, respectively. A total of 247, 195, and 204 embryos were examined for WT, XKO, and TKO irradiated with 10 Gy, respectively.

To further investigate the impact of H2AX or TH2A deletion on cell cycle progression in irradiated zygotes, we used live imaging to record the timing of the first cleavage following 0.5 and 10 Gy irradiation. In the absence of exogenous DNA damage, the cleavage curves of wild-type and H2AX- and TH2A-deleted zygotes showed a high degree of overlap. In all non-irradiated experimental groups, a small fraction of zygotes had initiated cell division at 14 HPI, and over 95% of zygotes entered the 2-cell stage by 17 HPI (Fig. 3A, B, Supplementary Movies 3–6), reinforcing the idea that the lack of H2AX or TH2A might have no effect on embryonic development under unstressed conditions. Moreover, the cleavage curves of wild-type and H2AX- and TH2A-deleted zygotes were right-shifted after exposure to 0.5 or 10 Gy irradiation, suggesting that the cleavage was delayed by the activation of G2 checkpoint as a process of DDR and that H2AX or TH2A deletion did not affect this process (Fig. 3A, B, Supplementary Movies 3–6). However, in H2AX-deleted zygotes, the resumption of cell cycle progression was significantly earlier, especially in the groups exposed to 10 Gy irradiation (Fig. 3A, B, Supplementary Movies 3–5), indicating that the maintenance of the irradiation-induced cell cycle arrest was impaired in the absence of H2AX. At 20 HPI, for instance, the completion rate of the first cleavage was 67.69% for H2AX-deleted zygotes, in contrast to only 33.60% for the wild-type zygotes and 26.47% for the TH2A-deleted ones (Fig. 3B), after the exposure to 10 Gy at G2 phase.

### Deletion of H2AX but not TH2A increases the micronucleus formation rate

The reduced cell cycle arrest in H2AX-deleted zygotes implies a shorter window for DNA repair, which may leave a substantial amount of DNA DSBs unrepaired. Untimely resolution of DSBs increases the risk of erroneous DNA repair and chromatin bridges [21]. Alternatively, unrepaired DNA lesions cause the generation of DNA fragments which may fail to be properly incorporated into the daughter nuclei and instead form micronuclei in the cytoplasm during the subsequent cell division. Therefore, the formation of chromatin bridges and micronuclei following the first cleavage was examined in the wild-type and H2AX- or TH2A-deleted zygotes that had been irradiated with 0.5 or 10 Gy at the G2 phase.

**Fig. 4:**
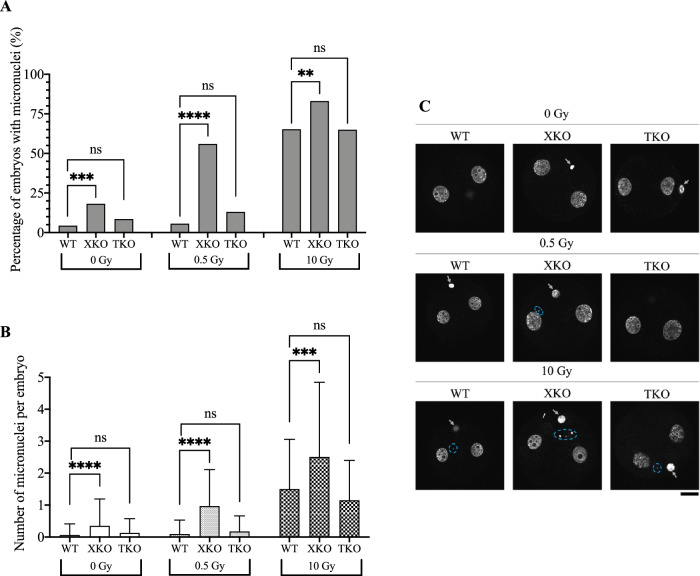
Effect of H2AX or TH2A deletion on micronucleus formation in irradiated zygotes after the first cleavage. **A** Percentages of embryos with micronuclei in their cytoplasm. Fisher’s exact test was used for statistical analysis (**P < 0.01; ***P < 0.001; ****P < 0.0001; ns, not significant). **B** Numbers of micronuclei in each embryo. Student’s *t*-test was used for statistical analysis (***P < 0.001; ****P < 0.0001; ns, not significant). **C** Representative Z-stack projections of embryos after the first cleavage. Micronulei are circled in blue. Gray arrows indicate polar bodies. For **A**–**C**, WT, XKO, and TKO represent wild-type, H2AX-deleted, and TH2A-deleted zygotes, respectively. More than four independent experiments were performed for each experimental condition. The data in **A** represent the cumulative results, whereas in **B** the average number of micronuclei per embryo was calculated. A total of 256, 77, and 94 embryos were examined for WT, XKO, and TKO irradiated with 0 Gy, respectively; a total of 232, 100, and 46 embryos were examined for WT, XKO, and TKO irradiated with 0.5 Gy, respectively; a total of 170, 71, and 60 embryos were examined for WT, XKO, and TKO irradiated with 10 Gy, respectively. Scale bar, 20 μm.

Consistent with our previous results, chromatin bridges were not observed in embryos irradiated with either 0.5 Gy (0/232) or 10 Gy (0/170) at the G2 phase of the first cell cycle. The deletion of H2AX or TH2A had no effect on chromatin bridge formation in embryos irradiated at the G2 phase. However, in the absence of H2AX, the percentage of embryos containing at least one micronucleus increased even without irradiation, likely due to endogenous DNA damage that occurs naturally within zygotes as the result of metabolic processes or other cellular activities (Fig. 4A). The irradiation of 0.5 Gy had no significant effect on micronucleus formation in wild-type and TH2A-deleted embryos but led to a dramatic rise of micronucleus formation rate in H2AX-deleted zygotes (Fig. 4A). Moreover, deletion of H2AX, not TH2A, significantly increased the number of micronuclei in each embryo (Fig. 4B, C). These results indicate that H2AX, but not TH2A, is involved in the DDR of zygotes. It was noted that the difference in the rate of irradiation-induced micronucleus formation between wild-type and H2AX-deleted embryos decreased in response to 10 Gy irradiation when compared to 0.5 Gy, presumably because, as described above, the extremely high level of DNA damage caused by such a high dose had overwhelmed the repair capacity of zygotes (Fig. 4A). These results provided evidence that TH2A was dispensable for DDR in zygotes whereas H2AX played an important role in post-irradiation recovery.

### Deletion of H2AX but not TH2A leads to a condensed chromatin structure

**Fig. 5:**
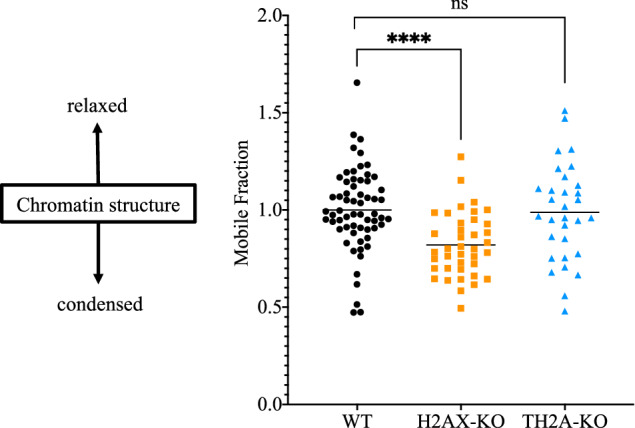
FRAP analysis of chromatin looseness in H2AX- or TH2A-deleted zygotes. The degree of chromatin looseness in each zygote was indicated by the value of the mobile fraction. The value of the mobile fraction in the wild-type zygotes was set at 1.0, and values for other conditions were calculated relative to this. Four independent experiments were conducted, with more than six zygotes analyzed in each condition per experiment. Student’s *t*-test was used for statistical analysis (****P < 0.0001; ns, not significant).

It remains unclear how H2AX is involved in DDR in zygotes, given the relatively low levels of induced γH2AX. It has been reported that, apart from its phosphorylated form γH2AX, H2AX per se also destabilizes nucleosomes in vitro [22]. It is possible that the high abundance of H2AX in zygotes contributes to DNA repair by the formation of a loose chromatin structure [23]. To confirm this, we examined the involvement of H2AX in chromatin relaxation in zygotes using fluorescent recovery after photobleaching (FRAP) with EGFP-H2B. A value known as the mobile fraction can be calculated from the fluorescence recovery rate after EGFP-H2B photobleaching; a high mobile fraction is thought to indicate a loosened chromatin structure, while a low value suggests a condensed one [24, 25]. TH2A deletion did not significantly affect the mobile fraction, but the value of the mobile fraction decreased significantly in H2AX-deleted zygotes (Fig. 5). These results suggest that the condensed chromatin structure resulting from H2AX deletion may pose a barrier for DNA repair proteins, thus hindering the repair process.

### Irradiation results in the phosphorylation of H2AX but not TH2A

**Fig. 6:**
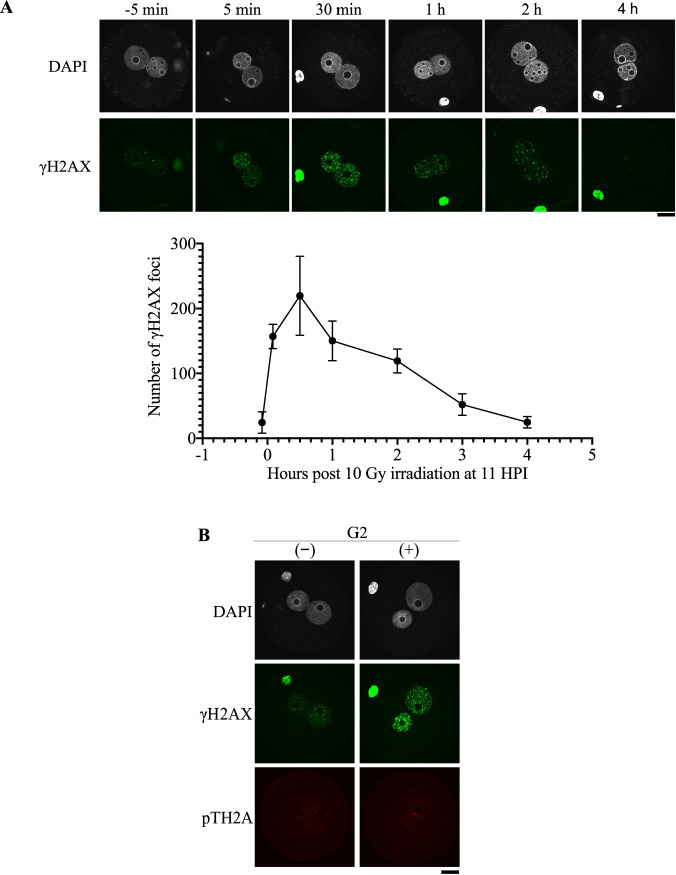
Phosphorylation of H2AX and TH2A post-irradiation. **A** γH2AX dynamics in zygotes irradiated with 10 Gy at 11 HPI (G2 phase). The upper panel shows representative images at each time point. Scale bar, 2 μm. Three independent experiments were performed. A total of 18, 17, 14, 14, 13, 9, and 14 embryos were examined for −5, 5, 30, 60, 120, 180, and 240 min post-irradiation, respectively. **B** Double immunostaining for γH2AX and TH2A phosphorylated at T127 in G2 phase zygotes, which were exposed to 0 or 10 Gy irradiation at 11 HPI and collected 30 min later. The symbols (−) and (+) represent nonirradiated and 10 Gy-irradiated zygotes, respectively. Scale bar, 20 μm.

H2AX is best known for the phosphorylation at its carboxyl end, resulting in γH2AX formation, which serves as a loading platform for various DDR-related proteins [2]. The removal of γH2AX by dephosphorylation after DNA repair is also crucial for cells to terminate DDR signaling and resume normal cellular processes [26]. To determine whether γH2AX was associated with zygotic DDR, γH2AX dynamics were examined over the period spanning from 5 min before irradiation to several hours post-irradiation. Following exposure to both 0.5 and 10 Gy, γH2AX foci formed within minutes after irradiation, and the number of foci began to decrease by 30 min post-irradiation (Figs. 6A and S4A). Combined with the results of micronucleus formation and preimplantation development, the increase and decrease in γH2AX signals suggest the formation and repair of DNA damage, respectively, which is analogous to what occurs in interphase somatic cells and GV oocytes after irradiation exposure [27, 28].

As opposed to the rapid formation of γH2AX, no increase in phosphorylated TH2A level was observed at G2 phase 30 min after irradiation (Fig. 6B). At M phase, TH2A was phosphorylated even without irradiation and the level of phosphorylated TH2A remained unchanged post-irradiation (Fig. S4B). This lack of response further supports the idea that TH2A is not directly involved in DDR.

### Phosphorylated CHK2 does not form foci in H2AX-deleted zygotes following irradiation

**Fig. 7:**
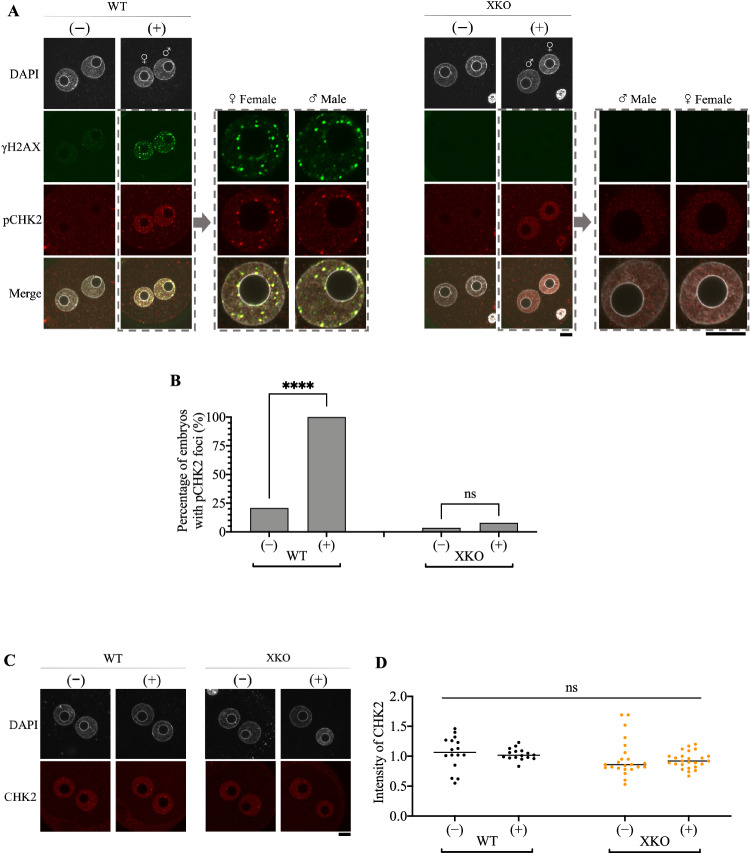
Immunostaining of γH2AX, CHK2 and pCHK2 post-irradiation. **A** Representative immunostaining images of γH2AX and pCHK2 in wild-type (WT) and H2AX-deleted (XKO) zygotes, which were exposed to 0 or 10 Gy irradiation at 11 HPI and collected 30 min later. The symbols (−) and (+) represent nonirradiated and 10 Gy-irradiated zygotes, respectively. The enlarged male (♂) and female (♀) pronuclei at the right sides of the original images show the foci formation of γH2AX and pCHK2 in irradiated embryos. **B** The percentage of embryos with pCHK2 foci in (**A**). The data represent the cumulative results of five independent experiments, with a total of 24 and 30 embryos examined for wild-type embryos irradiated with 0 and 10 Gy, respectively, and 30 and 38 embryos examined for H2AX-deleted embryos irradiated with 0 and 10 Gy, respectively. Fisher’s exact test was used for statistical analysis (****P < 0.0001; ns, not significant). **C** Representative immunostaining images of CHK2 in wild-type (WT) and H2AX-deleted (XKO) zygotes. The symbols (−) and (+) represent nonirradiated and 10 Gy-irradiated zygotes, respectively. **D** Quantitative analysis of CHK2 intensity in (**C**). The average fluorescence intensity of CHK2 for irradiated wild-type zygotes was set to 1.0, relative to which values for the other conditions were calculated. Three independent experiments were performed. In total, 16 and 16 embryos were examined for wild-type embryos irradiated with 0 and 10 Gy, respectively; 23 and 25 embryos were examined for H2AX-deleted embryos irradiated with 0 and 10 Gy, respectively. Student’s *t*-test was used for statistical analysis (ns, not significant). Scale bar, 20 μm.

Our previous research demonstrated that the activated form of CHK2, phosphorylated at T68 (pCHK2), was highly incorporated into the pronuclei of irradiated G2-phase zygotes, potentially contributing to cell cycle arrest [9]. Since colocalization of γH2AX and pCHK2 foci was found at DNA-damaged sites in somatic cells [29], double immunostaining for γH2AX and pCHK2 was performed on wild-type and H2AX-deleted zygotes following irradiation. γH2AX foci were only detected in wild-type zygotes and significantly elevated after irradiation (Fig. 7A). While an increase in pCHK2 level in the nucleoplasm was also found in H2AX-deleted zygotes as well as in the wild-type ones, pCHK2 foci were only formed in the wild-type ones (Fig. 7A, B). Enlarged images showed that foci of γH2AX and pCHK2 were mostly colocalized in both pronuclei of the wild-type zygote (Fig. 7A). The differential localization of pCHK2 was not due to changes in CHK2 expression levels, as neither H2AX deficiency nor irradiation affected CHK2 expression (Fig. 7C, D). These results suggest that for irradiated zygotes, γH2AX foci may be indispensable for the focus formation of pCHK2 and that the presence of pCHK2 foci is likely associated with the duration of the cell cycle arrest following the activation of DNA damage checkpoint.

## Discussion

Whereas the role of H2AX in the DDR of somatic cells has undergone extensive investigation, its specific function in early embryos remains unclear. This lack of clarity is further compounded by the paradox we previously identified: despite the high expression of H2AX in zygotes, the level of γH2AX after irradiation is relatively low [16, 17]. In the case of TH2A, the role of the SQ motif at its carboxyl end has yet to be explored. This study investigated the impact of deleting H2AX or TH2A on the DDR of preimplantation embryos exposed to two different doses of irradiation, i.e. 0.5 and 10 Gy, which cause a low and high level of DNA damage, respectively.

γH2AX has been reported to be directly involved in demethylation in somatic cells [30]. Additionally, research has highlighted the escalation of γH2AX levels in the paternal pronucleus during the progression of zygotes into the S phase [9, 31], which is thought to be associated with the active demethylation of the paternal genome [31]. However, our current results reveal that H2AX deletion has no discernible effect on preimplantation development. It is possible that γH2AX merely serves as a byproduct during active demethylation in the first cell cycle after fertilization, rendering Η2ΑΧ dispensable for the process per se. Alternatively, the impact of defective demethylation due to the absence of H2AX might only appear post-implantation, which is consistent with the fact that parthenogenetic embryos developed beyond the preimplantation stage [32].

The proper operation of the DNA damage checkpoint necessitates swift activation upon the formation of DNA damage, maintenance of cell arrest while the repair machinery attends to the damaged DNA and subsequent resumption of cell cycle progression upon successful DNA repair. Our live imaging results showed that the G2 arrest was promptly activated and effectively sustained in both wild-type and TH2A-deleted embryos. Furthermore, although the repair capacity of zygotes seemed to be overburdened by 10 Gy irradiation, exposure to 0.5 Gy γ-ray at the G2 phase neither induced micronucleus accumulation nor hindered preimplantation development in wild-type and TH2A-deleted embryos. This indicates the full functionality of the G2 checkpoint in the absence of TH2A.

In contrast to the unaffected G2 arrest in TH2A-deleted zygotes, H2AX deletion led to a shortened G2 arrest, consequently increasing the formation rate of micronuclei and compromising the formation rate of blastocysts, indicating that the DDR system was attenuated by H2AX deletion. CHK2 is a key component in checkpoint activation [33]. Yet there have been conflicting findings regarding the effect of CHK2 loss on G2 arrest following ionizing radiation: while one study using CHK2-KO mouse embryonic stem (ES) cells showed that CHK2 was dispensable for G2 arrest [34], another study suggested that CHK2 was not involved in the initiation of G2 arrest but played a role in its maintenance in mouse ES cells [35]. Our results showed that CHK2 was phosphorylated and activated in the absence of H2AX, which could contribute to efficient cell cycle arrest after irradiation in H2AX-deleted zygotes. However, without H2AX, CHK2 could not form foci, potentially contributing to the shortened cell cycle arrest.

A significant change in H2A variant composition might occur after the deletion of H2AX or TH2A, given their dominance among H2A variants at the 1-cell stage (Figure S1). Immunostaining analyses revealed that the loss of H2AX was compensated by TH2A and H2AZ but not by H2A or macro H2A, while all examined H2A variants, including H2AX, H2A, macroH2A, and H2AZ, compensated for the absence of TH2A (Figs. S5, S6). Given the established roles of H2AZ in DDR [2], it is possible that the increased deposition of H2AZ in the paternal pronucleus of H2AX-deleted zygotes also contributes to the changes in DDR. However, as the mRNA level of H2AZ is extremely low in zygotes (Fig. S1), its influence may be minor.

It is still unclear how zygotes sustain efficient DNA repair despite the relatively low levels of induced γH2AX. However, beyond H2AX phosphorylation, other H2AX modifications, such as acetylation and ubiquitination, also contribute to creating a chromatin structure favorable for DDR [36–39]. Moreover, our FRAP analysis provides evidence that H2AX may contribute to DDR by promoting a loosened chromatin structure, consistent with in vitro findings that H2AX destabilizes nucleosomes by impairing the binding of linker histone H1, which is involved in chromatin compaction and thus hinders the relaxation-dependent recruitment of DNA repair proteins [22, 40]. The removal of H1.2 (also known as H1c) from chromatin is crucial for ATM activation [41], and H1.2 expression is minimal in zygotes, beginning to increase only after the 4-cell stage [11]. Therefore, it is likely that in zygotes, the reduced levels of H1.2 lower the barrier to DDR, a process further enhanced by the abundant presence of H2AX, H3.3, and H1foo, which establishes an open chromatin structure that is accessible to various DDR proteins.

In recent years, there has been a growing interest in the alternative end joining (alt-EJ) repair pathway, which is implicated in mutagenesis during cancer development because it is less faithful than the two major DNA repair pathways in somatic cells: non-homologous end joining (NHEJ) and homologous recombination (HR) [42]. In nematode zygotes, irradiation-induced random DSBs in the paternal genome are predominantly repaired by the maternally provided error-prone alt-EJ and, notably, condensed chromatin prevents the use of the more faithful HR-dependent repair pathway, leading to high transgenerational lethality [43]. Zebrafish embryos show alt-EJ dominance in repairing DSBs induced by the CRISPR system [44]. Mutational signatures in mouse zygotes suggest that alt-EJ repairs a portion of CRISPR-induced DSBs [45, 46], while disruption of NHEJ or HR in mouse oocytes increases sperm-derived chromosomal aberrations [47]. There is no direct evidence yet for the dominant DNA repair pathway in mouse embryos. It remains unclear how the choice of DNA repair pathways and the resulting sequences at breakpoints are influenced by the relaxed chromatin structure in mouse zygotes, to which the abundant H2AX at least partially contributes.

Nevertheless, our results demonstrate that H2AX ensures adequate DNA repair when exposed to external damage, thus preventing micronucleus formation and embryo loss during the preimplantation period. In our experimental setup, we used heterozygous H2AX zygotes, with oocytes from H2AX-KO female mice and sperm from wild-type male mice because H2AX-KO male mice are infertile. Consequently, H2AX was only temporarily absent, appearing as the zygotes entered the 2-cell stage and coinciding with the major zygotic genome activation (ZGA). Nonetheless, this transient deficiency in H2AX significantly impacted zygotes exposed to irradiation, including micronucleus formation and disrupted preimplantation development. These findings underscore the critical role of the maternal genome in early development and highlight the risks associated with potential therapies on female infertility targeting components in DDR [48].

## Materials and methods

### Construction of H2AX- and TH2A-KO mice

We applied the CRISPR/Cas9 system for constructing H2AX- and TH2A-KO mice and generated vector constructs encoding the single guide RNAs. PCR products containing the T3 promoter–sgRNA–DraI target site were obtained using Ex Taq Hot Start Version (TaKaRa, RR006A) (Supplementary Table 1). With the TOPOTM TA CloningTM Kit (Cat# 450640, Thermo Fisher Scientific, MA, USA), the amplified PCR products were ligated into an empty vector lacking the T3 promotor. *E. coli* colonies containing the target DNA fragments were isolated, and the plasmids were extracted. The sequences of the target DNA fragments were confirmed by DNA sequencing (Eurofins Scientific, Grand Duchy of Luxembourg).

In vitro fertilization (IVF) and culture of embryos were conducted as previously reported [9]. Embryos were obtained via IVF with oocytes collected from female BDF1 and sperm collected from male ICR mice. Approximately five hours post-insemination (HPI), the embryos were microinjected with 10 ng/μl each of Cas9 cRNA and sgRNA targeting either H2AX or TH2A (Supplementary Table 1). The microinjected embryos were allowed to develop until the 2-cell stage in the incubator and were then transplanted into pseudopregnant female ICR. Founder mice (F0) were obtained either by natural birth or by uterine dissection 19 days post-transplantation. H2AX and TH2A mutant mice were selected from the offspring of F0 and B6J. Six generations of backcrossing were performed to bring the genetic background of mutant mice closer to B6J.

However, during the process of backcrossing, we observed a significant reduction in the birth rate of H2AX-KO mice with a B6J background (unpublished data). Therefore, after six generations of backcrossing, female H2AX-KO mice were mated with wild-type male DBA/2 mice to obtain H2AX mutant mice with a BDF1 genetic background. H2AX heterozygous mutant male and female mice with a BDF1 genetic background were then mated to produce H2AX homozygous mutant mice, whose birth rate conformed to Mendel’s laws. Although the birth rate of TH2A homozygous mutant mice was not significantly affected by the genetic background, TH2A-KO mice were bred in the same way as H2AX-KO mice to keep the experimental condition consistent. These KO mice were used in subsequent analyses.

All procedures involving animals underwent review and received approval from the University of Tokyo Institutional Animal Care and Use Committee. The execution of these procedures adhered to the Guiding Principles for the Care and Use of Laboratory Animals.

### Extraction of genomic DNA and confirmation of genotype by electrophoresis

A portion (approximately 5 mm) of the mouse tail was excised and added to a solution consisting of 100 μl of tail lysis buffer (0.05 M Tris–HCl, 0.063% sodium dodecyl sulfate, 0.02 M NaCl, 0.001 M EDTA) and 1 μl of proteinase K (Takara Bio Inc., Shiga, Japan) in a 1.5 ml tube. The solution was then incubated at 55 °C with shaking for at least 2 h to ensure complete dissolution. The mixture was centrifuged at 16,000 rpm for 10 min at room temperature. The supernatant was transferred to a new 1.5 ml tube and thoroughly mixed with 100 μl of phenol–chloroform–isoamyl alcohol (Nippon gene., Osaka, Japan). After centrifuging at 16,000 rpm for 10 min at room temperature, the upper layer was transferred to another new 1.5 ml tube and mixed with 5 μl of 3 M sodium acetate and 250 μl of 100% ethanol, followed by centrifugation at 16,000 rpm for 10 min. The liquid layer was removed, and the DNA precipitate was washed with 70% ethanol. Subsequently, the liquid layer was removed, and the precipitate was dissolved in sterile water to a final concentration of 100 μg/μl.

For the detection of wild-type and mutant H2AX, PCR was conducted on the extracted genomic DNA using primers designed to flank the deletion region (Supplementary Table 1) and GoTaq® Green Master Mix. Subsequent electrophoresis allowed genotype determination by comparison of the resulting bands.

For the detection of wild-type and mutant TH2A, PCR products (Supplementary Table 1) were digested with the restriction enzyme TasI (FD1354, Thermo Fisher Scientific) to generate fragments before electrophoresis was performed.

### IVF, γ-irradiation and examination of micronuclei

Protocols for IVF, γ-irradiation, and the examination of micronuclei followed those described in a previous study [9].

### Immunostaining

Embryos were fixed with 3.7% PFA and 0.2% Triton X100 in PBS, then washed three times with 0.1% BSA in PBS. The embryos were then placed in a primary antibody diluted in 1% BSA and 0.2% Tween in PBS at 4 °C for 16 h. After being washed three times with 0.1% BSA in PBS, the embryos were incubated with the secondary antibody diluted in 1% BSA and 0.2% Tween in PBS at room temperature for 1 h (see Supplementary Table 2 for detailed information on each antibody). The embryos were finally washed and mounted on a sliding glass using a VECTASHILD mounting medium with DAPI (4’,6-diamidino-2-phenylindole) (#H-1200, Funakoshi).

### Live imaging

All live imaging was performed using a CytoSMART^TM^ LUX2 device (CytoSMART Technologies). The results were independently observed by YW and a research assistant, with the final outcomes being collectively summarized. The assistant was blinded to the group allocation during the observation.

### Image shooting and processing

All immunostaining images were taken on a confocal laser scanning microscope FV3000 (OLYMPUS) and analyzed by ImageJ.

### Fluorescent recovery after photobleaching (FRAP)

Zygotes were microinjected with 500 ng/μl EGFP-H2B cRNA at 3 HPI. At 9 HPI, the microinjected zygotes were transferred to a glass bottom dish (CELL view, Cell Culture Dish, Four Components, Greiner Bio-One) containing KSOM-HEPES medium that had been pre-incubated at 38 °C and 5% CO_2_ for 20 min and covered with mineral oil. FRAP analysis was conducted using the confocal laser microscope equipped with a ×64 oil immersion lens, with the sample stage preheated to 38 °C by a Microscope Incubate System (Tokai Hit, Co.). FV31S-DT (Olympus Corporation) was used to set the GFP fluorescence photobleaching region (ROI: region of interest), the non-photobleachable region (REF: reference), and the region without GFP fluorescence (BG: background). The excitation of the 488 nm laser was set to 0.4% under normal conditions, and the fluorescence intensity was adjusted to be around 2000. Three photos were taken every 5 s before photobleaching. The photobleaching was performed for 1 s by setting the argon laser excitation to 3%. Subsequently, another ten photos were taken every 5 s. The fluorescence intensities of ROI, REF, and BG were quantified from these images using ImageJ. The value from ROI minus that from BG was divided by the value from REF minus that from BG. The relative value was calculated by dividing the value at each time point by the average of the three values before photobleaching. The bleaching rate was calculated by subtracting the value immediately after bleaching from the average of the three values before bleaching. The recovery rate was calculated by subtracting the value immediately after bleaching from the value at the final time point. The value of the mobile fraction was calculated by dividing the recovery rate by the bleaching rate [49–51].

### Supplementary information

Supplementary Table 1

Supplementary Table 2

Legends for Supplemental Figures

Supplemental Figure S1

Supplemental Figure S2

Supplemental Figure S3

Supplemental Figure S4

Supplemental Figure S5

Supplemental Figure S6

Supplementary Movie 1

Supplementary Movie 2

Supplementary Movie 3

Supplementary Movie 4

Supplementary Movie 5

Supplementary Movie 6

## Data Availability

All data that support the findings of this study can be made available from the corresponding authors upon reasonable request.
