# Supplementary material for: Involvement of H2A variants in DNA damage response of zygotes
Source: Cell Death Discov. 2024 May 14;10:231. doi: 10.1038/s41420-024-01999-0 (PMC11094039; doi:10.1038/s41420-024-01999-0)
Supplement: Supplementary file 1 — Supplementary Table 1 [file 41420_2024_1999_MOESM1_ESM.docx]

**Supplementary Table 1. Sequences used in this study.**

| **Vector construction** |  |
| --- | --- |
| Forward primer for H2AX | GAAATTAACCCTCACTAAAGGGAACTACGCACAACGAATGTTTTAGAGCTAGAAATAGC |
| Forward primer for TH2A | GAAATTAACCCTCACTAAAGGTACTCGAGCACCGCTGCCGTTTTAGAGCTAGAAATAGC |
| Common reverse primer | TTTAAAAAAGCACCGACTCGGTGCCACTTTTTCAAGTTGATAACGGACTAGCCTTATTTTAACTTGCTATTTCTAGCTCTAAAAC |
|  | |
| **Single guide RNA (sgRNA)** | |
| H2AX | TACTCGAGCACCGCTGCC |
| TH2A | GAACTACGCACAACGAAT |
|  | |
| **Genotype confirmation.** | |
| Forward primer for H2AX | GCTGCTGCGGAAAGGCC |
| Reverse primer for H2AX | TCAGTACTCCTGAGAGGCCTG |
| Forward primer for TH2A | TCCGGCATGGCTCAAGTAACC |
| Reverse primer for TH2A | CTGGATGTTGGGCAGGACGC |
