# Supplementary material for: Involvement of H2A variants in DNA damage response of zygotes
Source: Cell Death Discov. 2024 May 14;10:231. doi: 10.1038/s41420-024-01999-0 (PMC11094039; doi:10.1038/s41420-024-01999-0)
Supplement: Supplementary file 2 — Supplementary Table 2 [file 41420_2024_1999_MOESM2_ESM.docx]

**Supplementary Table 2. Antibodies and applied concentrations.**

| **Name and Origin** | **Concentration** | **Catalog No. and Manufacturer details** |
| --- | --- | --- |
| Anti-H2AX,  rabbit, polyclonal | 1:500 | # 20669, Abcam PLC, UK |
| Anti-TH2A,  rabbit, polyclonal | 1:2000 | Produced and kindly provided by Dr. Shinagawa [18] |
| Anti-H2AX phosphorylated at S139, mouse, monoclonal | 1:1000 | #05-636, clone JBW301, Sigma-Aldrich, USA |
| Anti-TH2A phosphorylated at T127, rabbit, polyclonal | 1:500 | Produced and kindly provided by Dr. Okada [19] |
| Anti-H2A,  mouse, monoclonal | 1:100 | #D210-3, cloneC10037, MBL, Japan |
| Aniti-macroH2A,  rabbit, polyclonal | 1:100 | #07-219, EMD Millipore Corporation, USA |
| Anyi-H2AZ,  rabbit, polyclonal | 1:200 | #07-594, Sigma-Aldrich, Australia |
| Anti-CHK2 phosphorylated at T68, rabbit, polyclonal | 1:100 | #2661, Cell Signaling, USA |
| Alexa Fluor 488-labeled goat anti-mouse IgG secondary antibody | 1:100 | #A11001, Invitrogen, USA |
| Alexa Fluor 647-labeled donkey anti-rabbit IgG secondary antibody | 1:100 | #A31573, Invitrogen, USA |
