# Supplementary material for: Involvement of H2A variants in DNA damage response of zygotes
Source: Cell Death Discov. 2024 May 14;10:231. doi: 10.1038/s41420-024-01999-0 (PMC11094039; doi:10.1038/s41420-024-01999-0)
Supplement: Supplementary file 3 — Legends for Supplemental Figures [file 41420_2024_1999_MOESM3_ESM.docx]

**Supplementary figure legends**

**Figure S1. Relative mRNA levels of H2A variants in GV oocytes and preimplantation embryos**

With RNA-sequencing data [18], the expression levels of mRNA for TH2A, H2AX, H2A, macroH2A, and H2AZ were analyzed at different stages, including GV oocyte, 1-cell (zygote), 2-cell, 4-cell, morula, and blastocyst stages.

**Figure S2. Deletion of H2AX or TH2A gene using CRISPR/Cas9**

**A.** and **B.** Confirmation of H2AX or TH2A deletion by electrophoresis. WT, H and KO represent wild-type, heterogeneous knockout, and homogeneous knockout, respectively. **C.** Construction of H2AX- or TH2A-deleted zygotes by *in vitro* fertilization of H2AX- or TH2A-KO oocytes and wild-type sperm. **D.** and **E.** Immunostaining to confirm the absence of H2AX (D) or TH2A (E) protein in zygotes generated as shown in C and collected at 11 HPI. Scale bar, 20 μm.

**Figure S3. The deposition of H2AX and TH2A in preimplantation embryos**

**A.** Immunostaining of H2AX at different stages of the preimplantation period. **B.** Immunostaining of TH2A at different stages of the preimplantation period. **C.** Immunostaining of H2AX in M phase zygotes. **D.** Immunostaining of γH2AX in M phase zygotes with or without 10 Gy irradiation. In all figures, H2AX-KO and TH2A-KO indicate embryos derived from the fertilization of wild-type sperm and oocytes with the corresponding gene knockouts. For A and B, embryos were collected for immunostaining at 11, 28, 42, 66, and 96 HPI for 1-cell, 2-cell, 4-cell, morula, and blastocyst stage embryos, respectively. For C, zygotes were cultured until 12 HPI, moved to nocodazole-containing media to arrest at M phase, and then collected for immunostaining at 15 HPI. For D, zygotes were cultured until 12 HPI, moved to nocodazole-containing media to arrest at M phase, exposed to 0 (−) or 10 Gy (+) irradiation at 15 HPI, and then collected 30 min later for immunostaining. Scale bar, 20 μm.

**Figure S4. Phosphorylation of H2AX and TH2A post-irradiation**

**A.** γH2AX dynamics in zygotes irradiated with 0.5 Gy at 11 HPI (G2 phase). The upper panels show representative images at each time point. Three independent experiments were performed. A total of 8, 12, 16, 12, and 12 embryos were examined for -5, 5, 30, 60, and 120 min post-irradiation, respectively. **B.** Double immunostaining for γH2AX and TH2A phosphorylated at T127 in M phase zygotes. Zygotes were cultured until 12 HPI, moved to nocodazole-containing media to arrest at M phase, exposed to 0 (−) or 10 Gy (+) irradiation at 15 HPI, and then collected 30 min later for immunostaining.

**Figure S5. Impact of H2AX deletion on the nuclear deposition of each H2A variant in zygotes**

Each H2A variant, including **A.** H2AX, **B.** TH2A, **C.** H2A, **D.** macroH2A, and **E.** H2AZ, was detected using immunostaining in the two pronuclei of wild-type or H2AX-deleted zygotes at 11 HPI. The average fluorescence intensity of H2A variants for wild-type maternal pronucleus was set to 1.0, relative to which values for the other conditions were calculated. Three independent experiments were performed, with more than six zygotes used each time. Student’s t-test was used for statistical analysis ((**, P < 0.01; ****, P < 0.0001; ns, not significant). Scale bar, 20 μm.

**Figure S6. Impact of TH2A deletion on the nuclear deposition of each H2A variant in zygotes**

Each H2A variant, including **A.** TH2A, **B.** H2AX, **C.** H2A, **D.** macroH2A, and **E.** H2AZ, was detected using immunostaining in the two pronuclei of wild-type or TH2A-deleted zygotes at 11 HPI. The average fluorescence intensity of H2A variants for wild-type maternal pronucleus was set to 1.0, relative to which values for the other conditions were calculated. Three independent experiments were performed, with more than six zygotes used each time. Student’s t-test was used for statistical analysis ((**, P < 0.01; ***, P < 0.001; ****, P < 0.0001; ns, not significant). Scale bar, 20 μm.
