# Supplementary figures and images for: Involvement of H2A variants in DNA damage response of zygotes
Source: Cell Death Discov. 2024 May 14;10:231. doi: 10.1038/s41420-024-01999-0 (PMC11094039; doi:10.1038/s41420-024-01999-0)

Figure S1

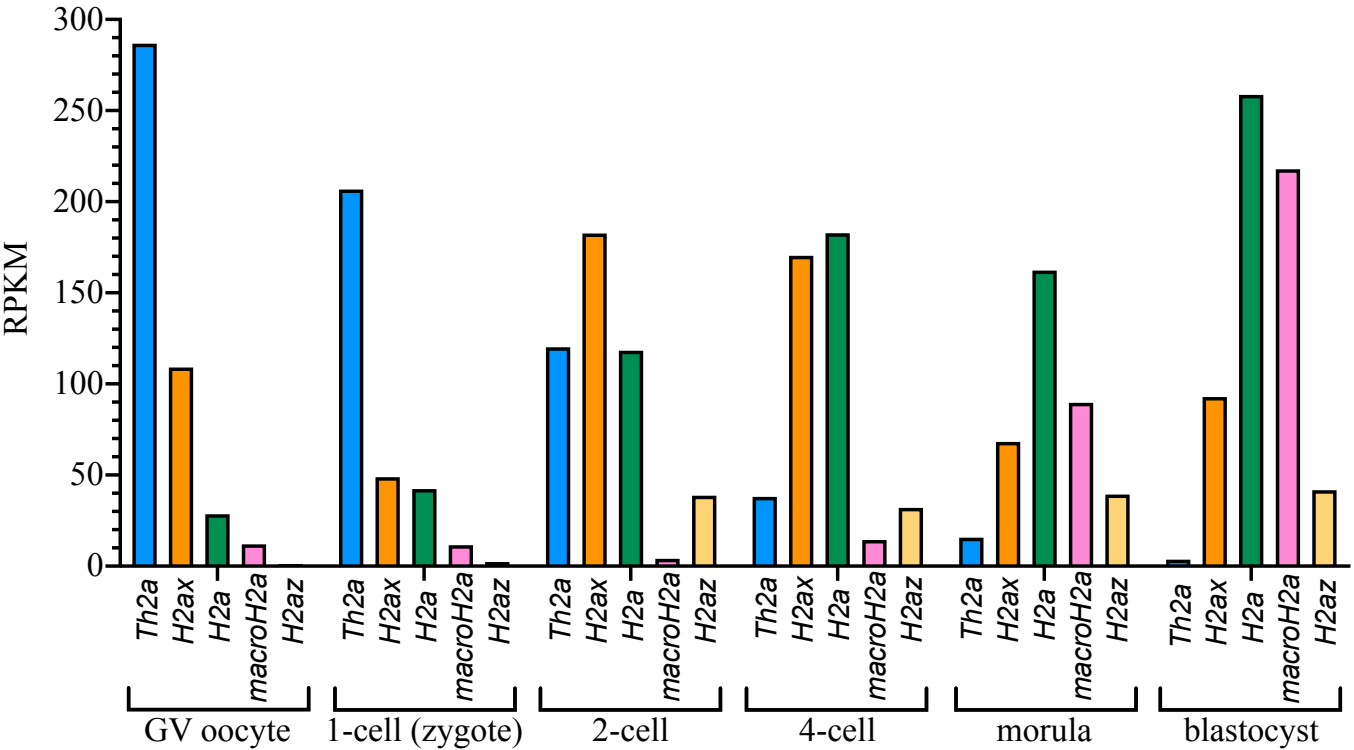

Supplement: Supplementary file 4 — Supplemental Figure S1 [file 41420_2024_1999_MOESM4_ESM.pdf]

**Figure S2**

**A**

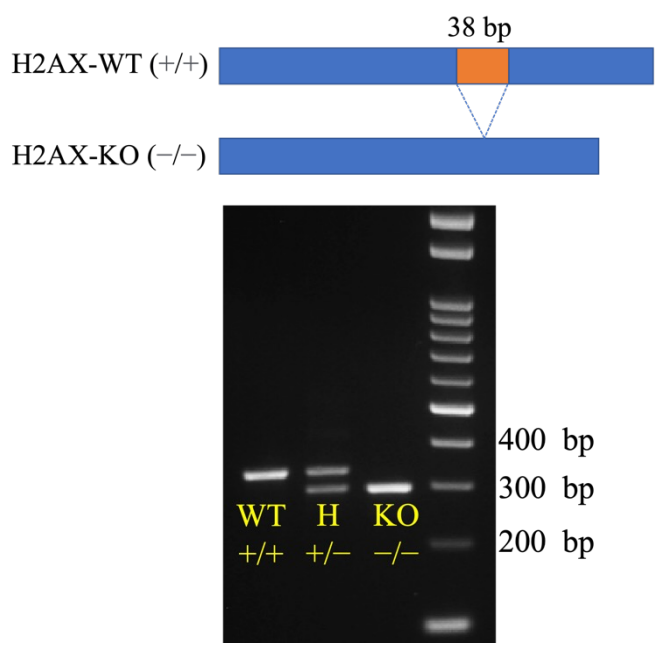

**B**

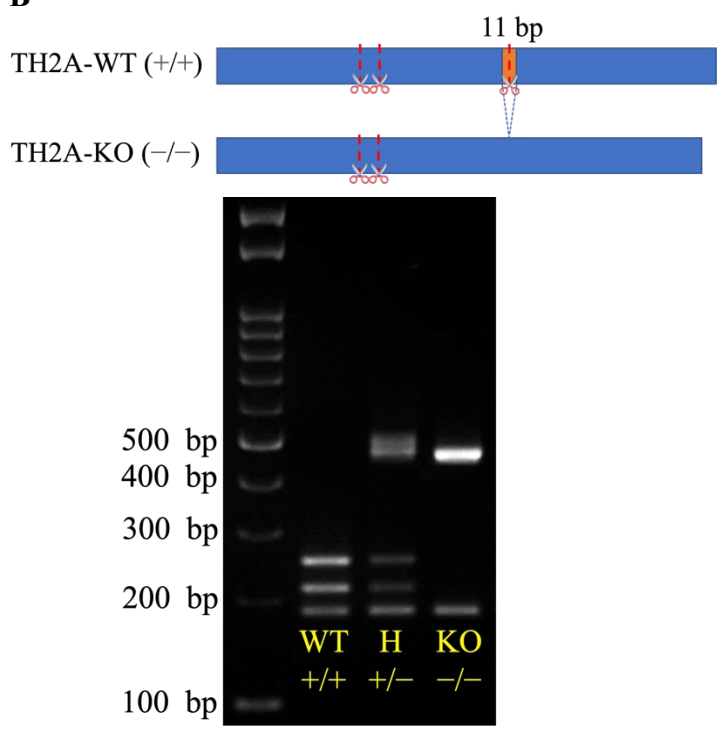

**C**

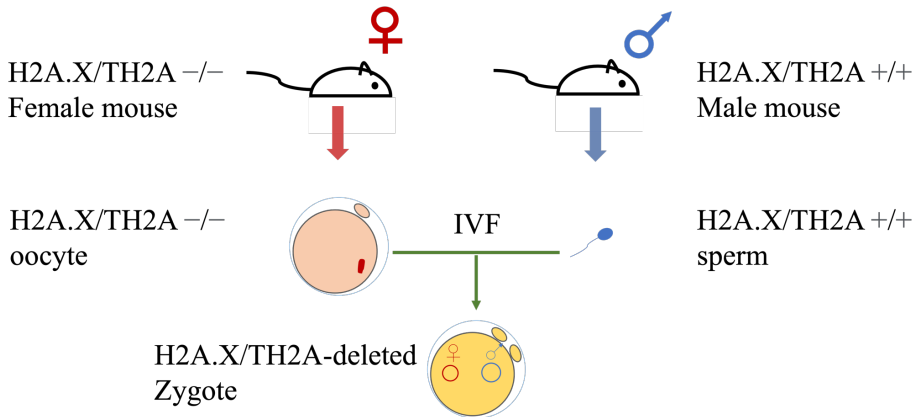

**D**

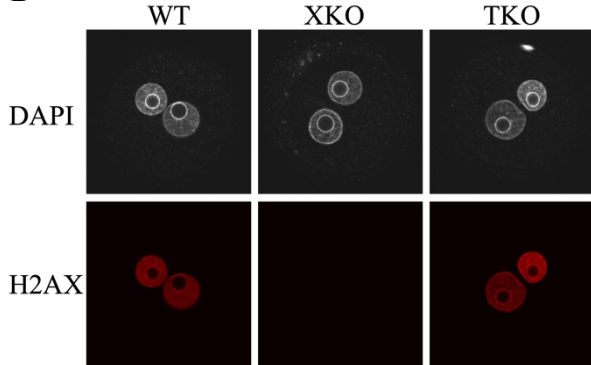

**E**

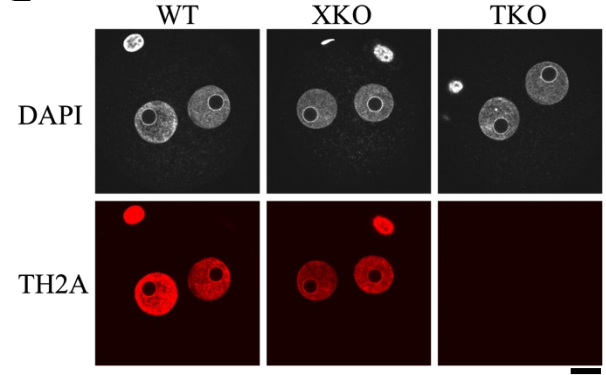

Supplement: Supplementary file 5 — Supplemental Figure S2 [file 41420_2024_1999_MOESM5_ESM.pdf]

Figure S3

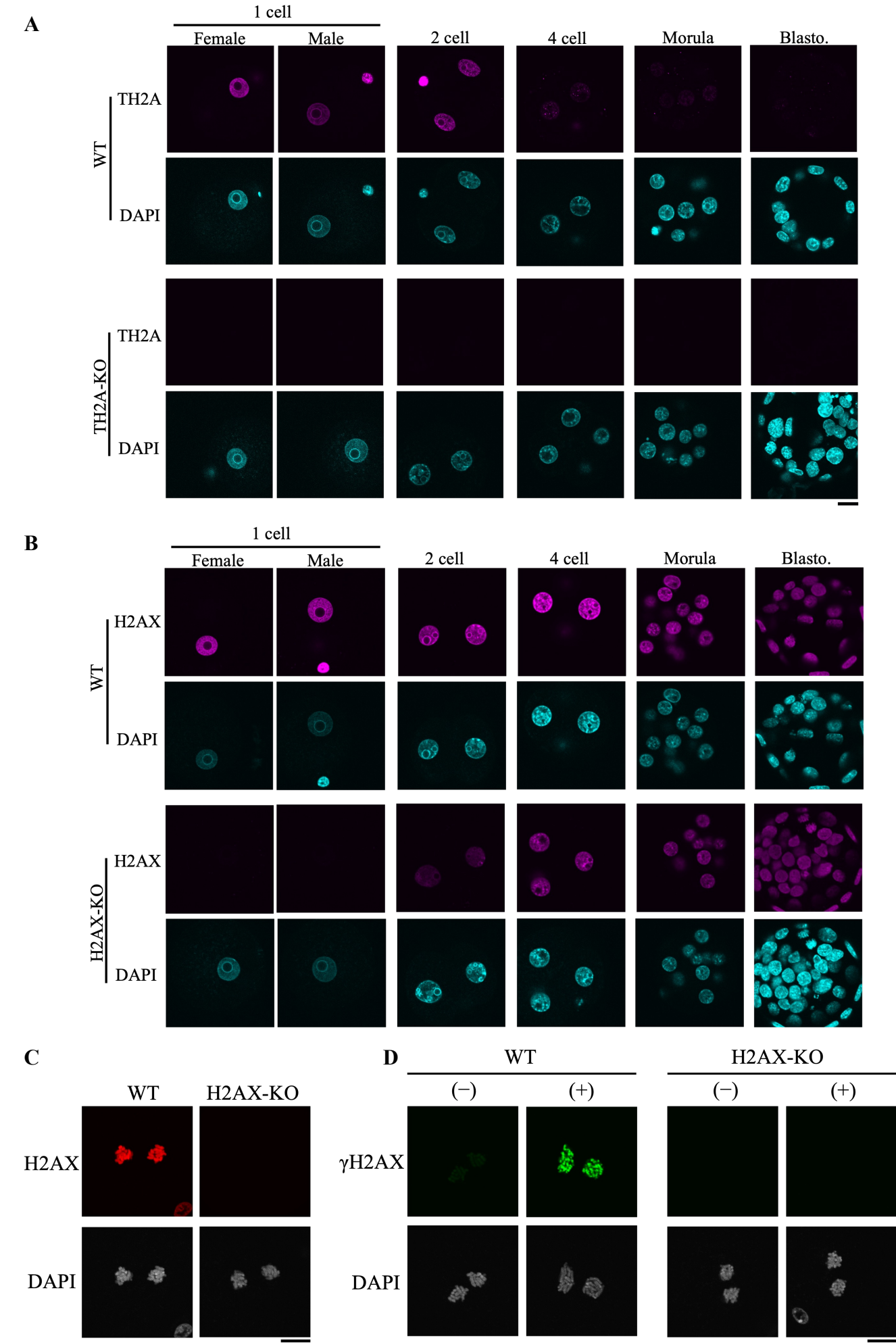

Supplement: Supplementary file 6 — Supplemental Figure S3 [file 41420_2024_1999_MOESM6_ESM.pdf]

**Figure S4**

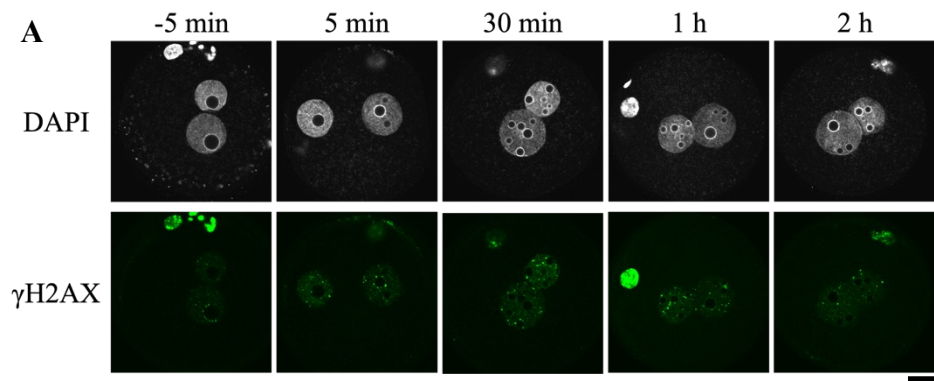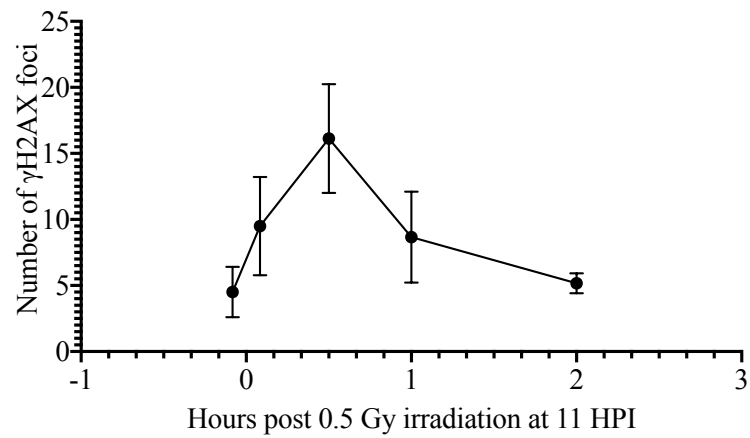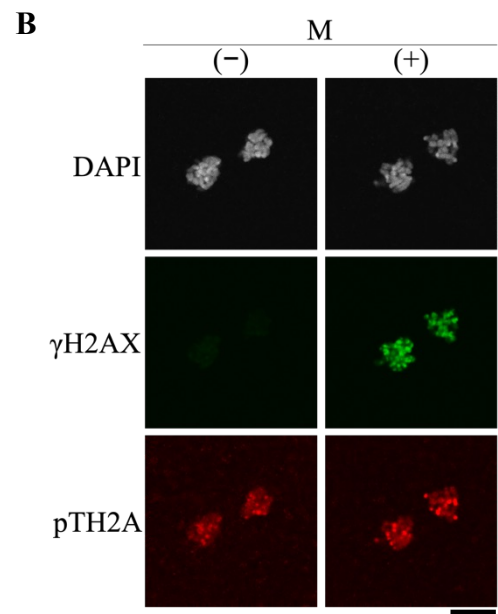

Supplement: Supplementary file 7 — Supplemental Figure S4 [file 41420_2024_1999_MOESM7_ESM.pdf]

Figure S5

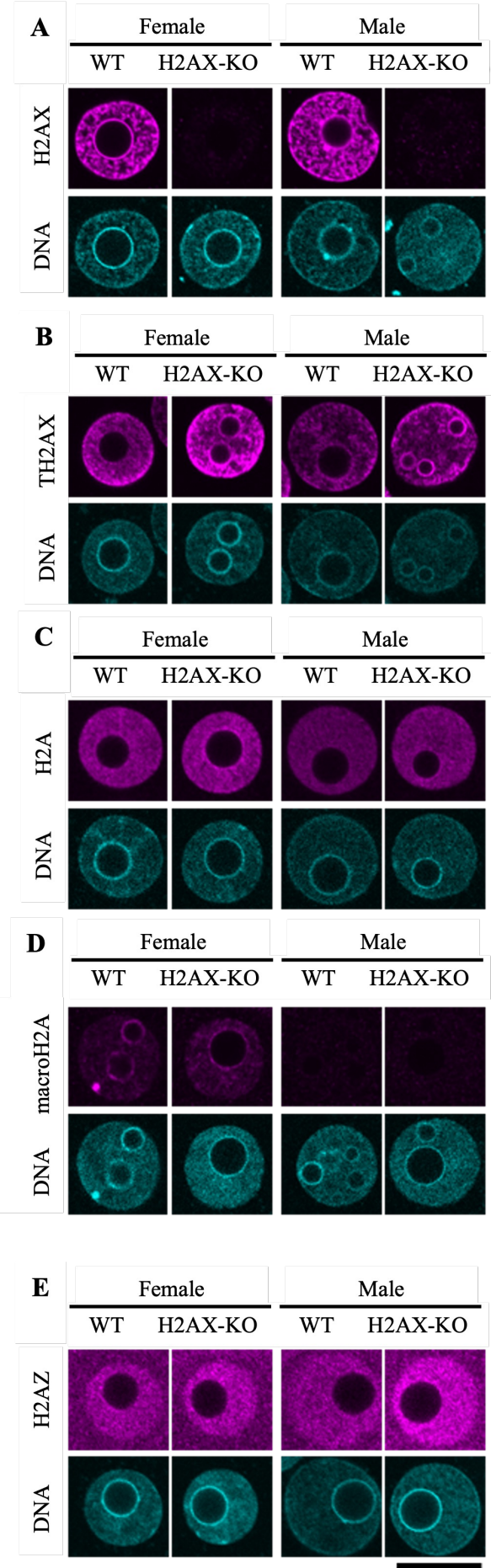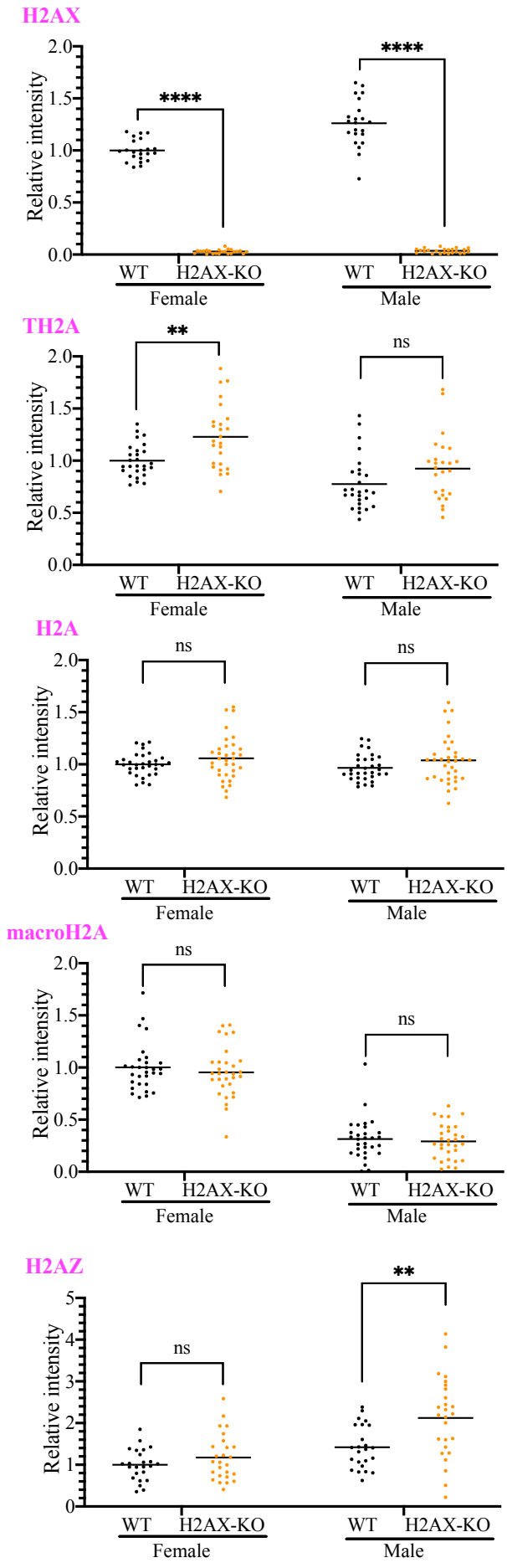

Supplement: Supplementary file 8 — Supplemental Figure S5 [file 41420_2024_1999_MOESM8_ESM.pdf]

Figure S6

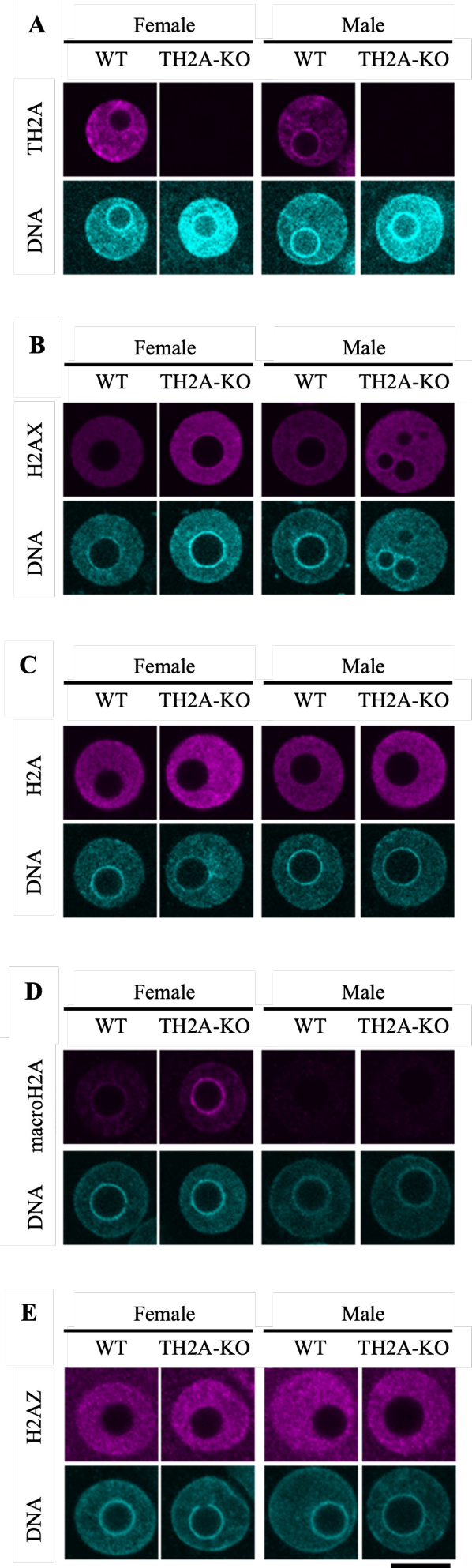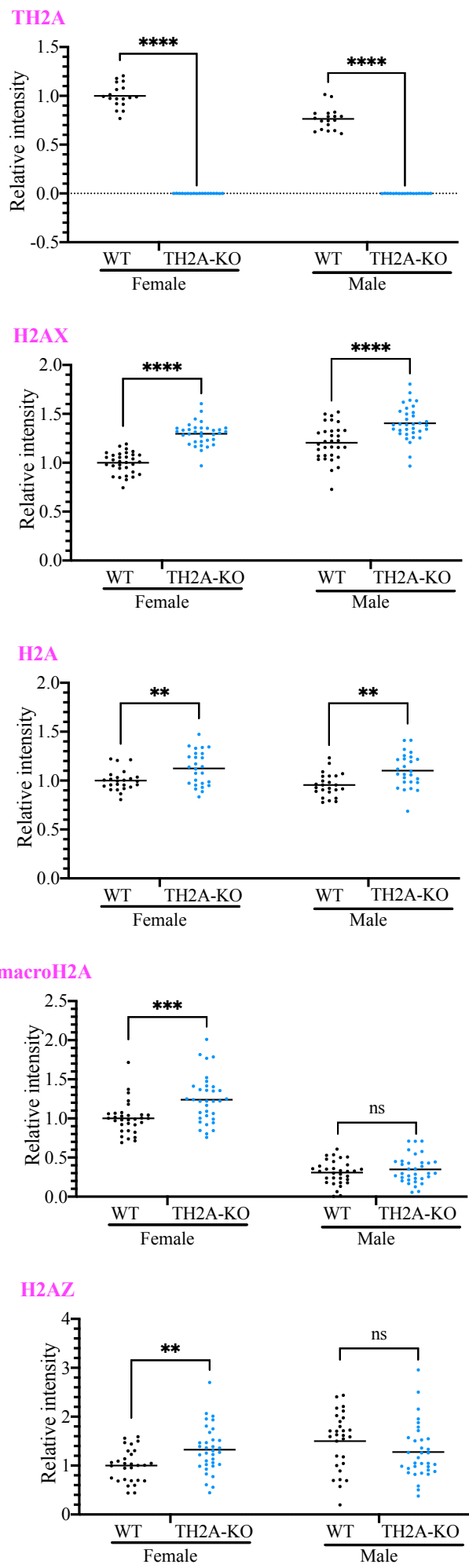

Supplement: Supplementary file 9 — Supplemental Figure S6 [file 41420_2024_1999_MOESM9_ESM.pdf]
